# Meetings

**Published:** 1898-07

**Authors:** 


### Meetings.

The regular meeting of the Tri-State Dental Association of Michigan, Indiana and Ohio was held in Hotel Victory, June 21st, 22d, and 23d. The program was carried out fully; everything went smoothly from beginning to end.

This body has scarcely any routine business to occupy the time, so it was wholly given to practical and scientific matters, except so far as the social feature was indulged. There were many good papers read, and well discussed. These we hope to give in future numbers of The Register. The social feature of the occasion was of the most delightful kind, the members were all thrown together for the whole time, little or no outside attractions, and every one seemed disposed to make all possible of this opportunity.

Many old acquaintances were renewed and strengthened, and many new acquaintances and friendships were made.

There were present between 450 and 500. The place proved to be a delightful one for such a meeting, and every one enjoyed himself to the full.

